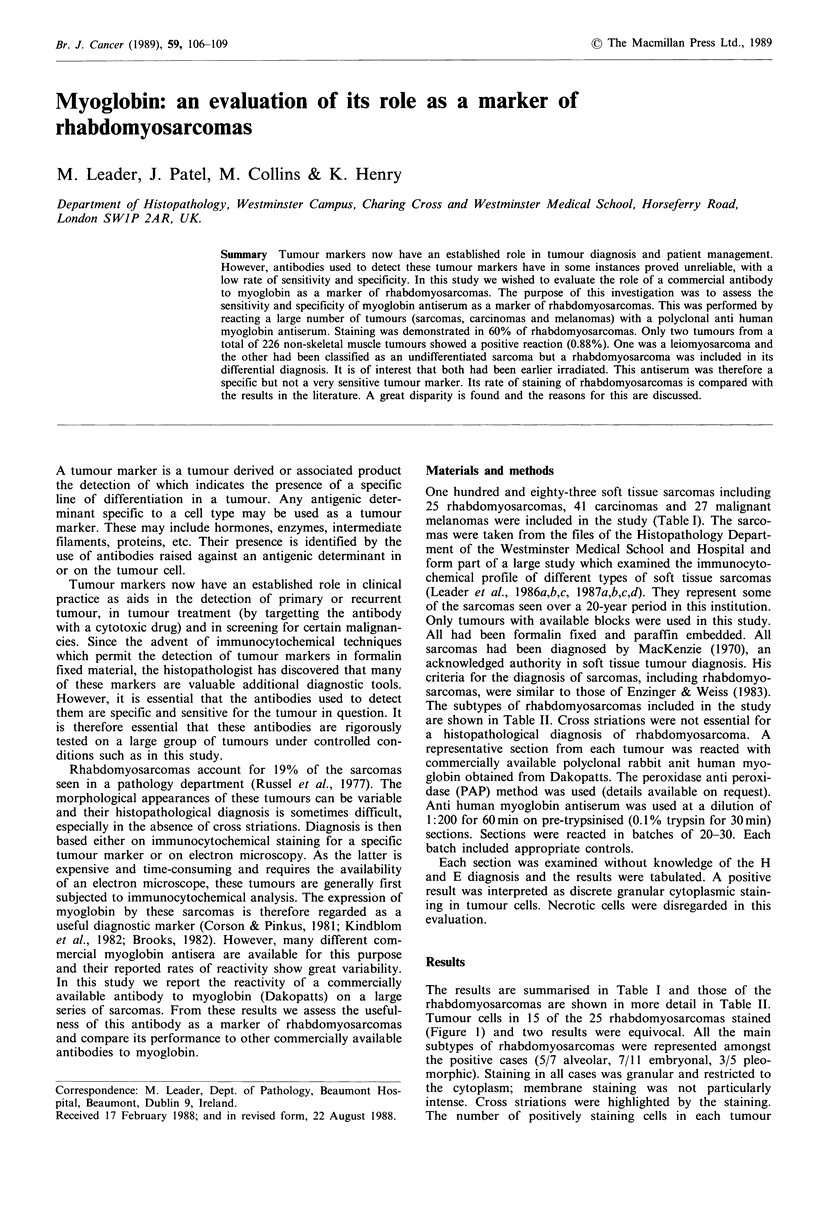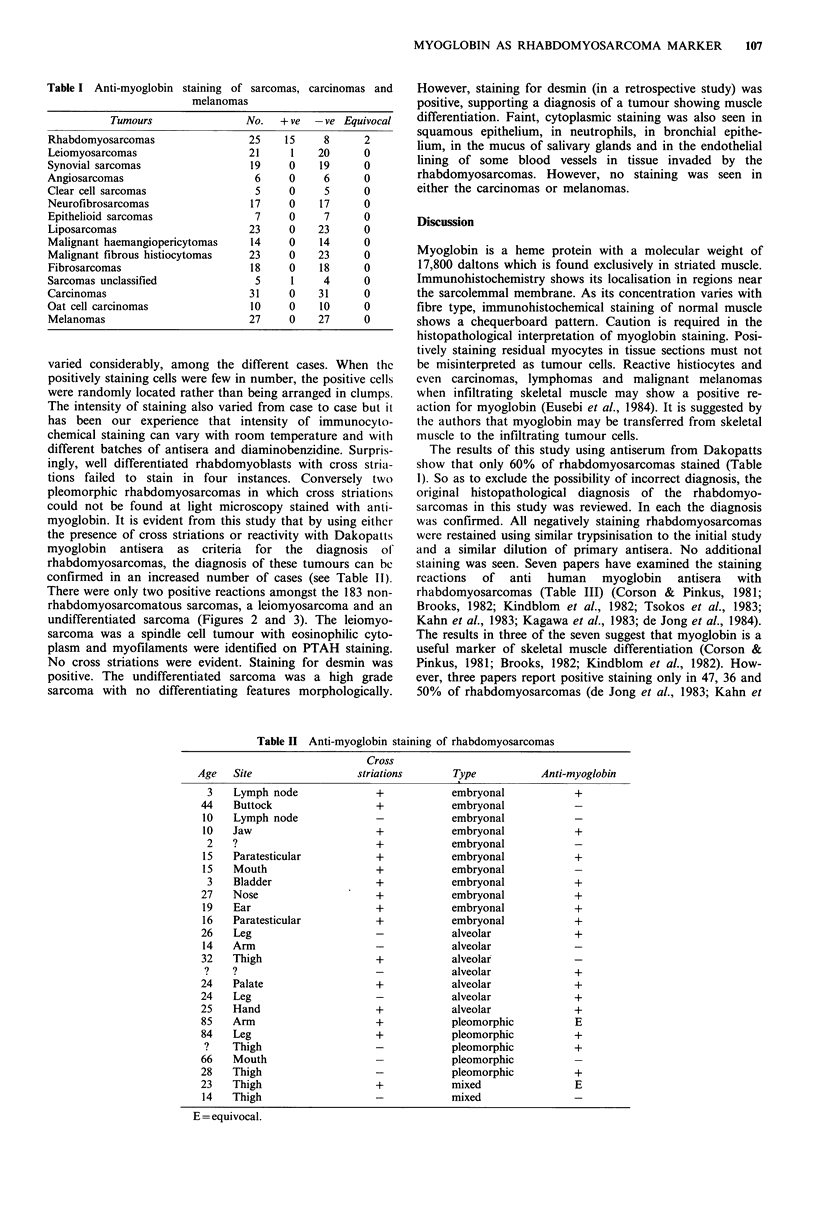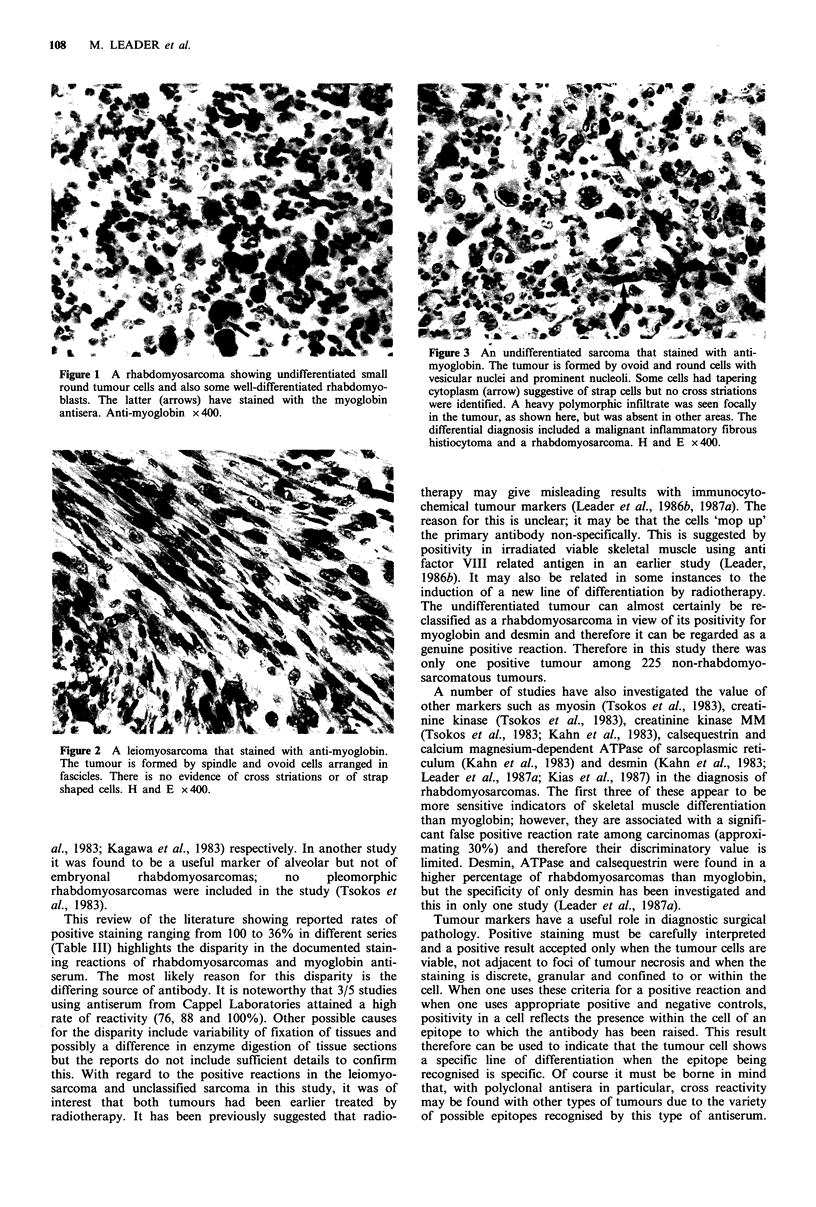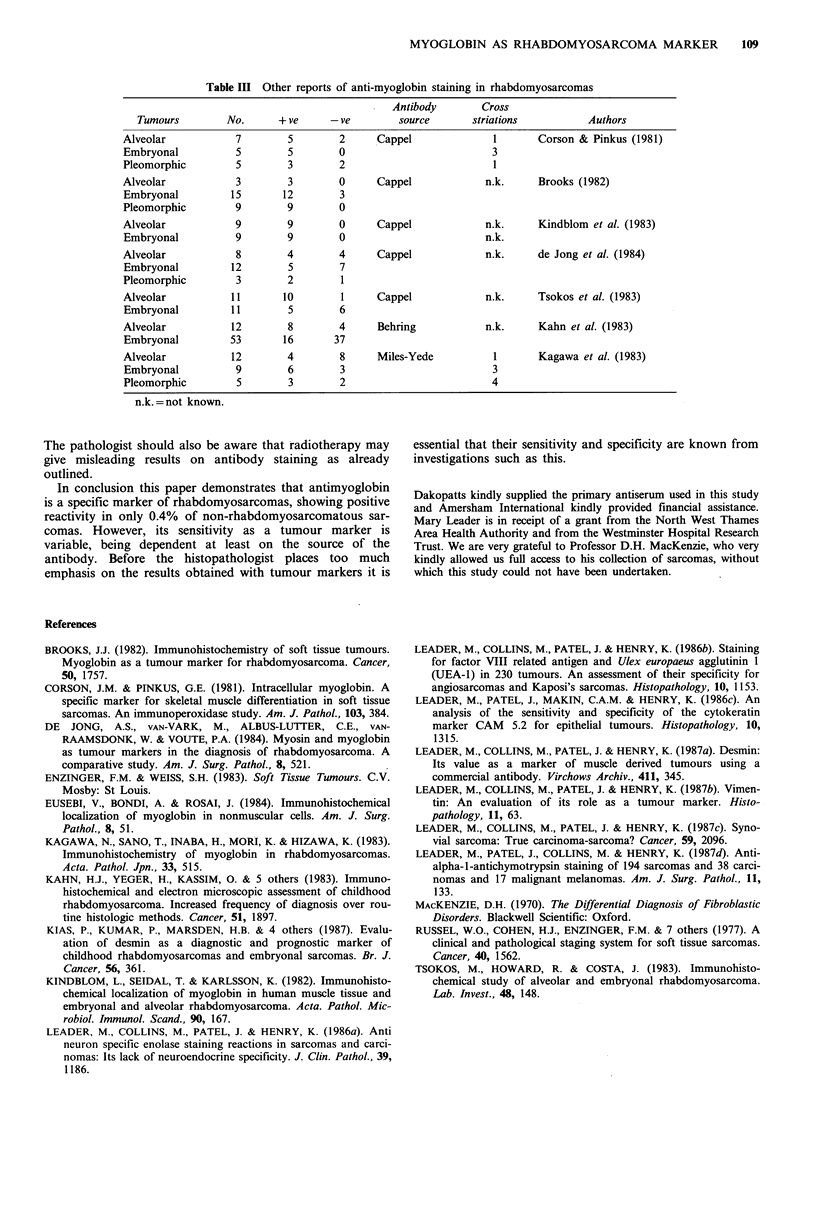# Myoglobin: an evaluation of its role as a marker of rhabdomyosarcomas.

**DOI:** 10.1038/bjc.1989.21

**Published:** 1989-01

**Authors:** M. Leader, J. Patel, M. Collins, K. Henry

**Affiliations:** Department of Histopathology, Charing Cross and Westminster Medical School, London, UK.

## Abstract

**Images:**


Br. J. Cancer (1989), 59, 106–109

© The Macmillan Press Ltd., 1989

### Myoglobin: an evaluation of its role as a marker of rhabdomyosarcomas

M. Leader, J. Patel, M. Collins & K. Henry

*Department of Histopathology, Westminster Campus, Charing Cross and Westminster Medical School, Horseferry Road, London SW1P 2AR, UK.*

**Summary** Tumour markers now have an established role in tumour diagnosis and patient management. However, antibodies used to detect these tumour markers have in some instances proved unreliable, with a low rate of sensitivity and specificity. In this study we wished to evaluate the role of a commercial antibody to myoglobin as a marker of rhabdomyosarcomas. The purpose of this investigation was to assess the sensitivity and specificity of myoglobin antiserum as a marker of rhabdomyosarcomas. This was performed by reacting a large number of tumours (sarcomas, carcinomas and melanomas) with a polyclonal anti human myoglobin antiserum. Staining was demonstrated in 60% of rhabdomyosarcomas. Only two tumours from a total of 226 non-skeletal muscle tumours showed a positive reaction (0.88%). One was a leiomyosarcoma and the other had been classified as an undifferentiated sarcoma but a rhabdomyosarcoma was included in its differential diagnosis. It is of interest that both had been earlier irradiated. This antiserum was therefore a specific but not a very sensitive tumour marker. Its rate of staining of rhabdomyosarcomas is compared with the results in the literature. A great disparity is found and the reasons for this are discussed.

A tumour marker is a tumour derived or associated product the detection of which indicates the presence of a specific line of differentiation in a tumour. Any antigenic determinant specific to a cell type may be used as a tumour marker. These may include hormones, enzymes, intermediate filaments, proteins, etc. Their presence is identified by the use of antibodies raised against an antigenic determinant in or on the tumour cell.

Tumour markers now have an established role in clinical practice as aids in the detection of primary or recurrent tumour, in tumour treatment (by targetting the antibody with a cytotoxic drug) and in screening for certain malignancies. Since the advent of immunocytochemical techniques which permit the detection of tumour markers in formalin fixed material, the histopathologist has discovered that many of these markers are valuable additional diagnostic tools. However, it is essential that the antibodies used to detect them are specific and sensitive for the tumour in question. It is therefore essential that these antibodies are rigorously tested on a large group of tumours under controlled conditions such as in this study.

Rhabdomyosarcomas account for 19% of the sarcomas seen in a pathology department (Russel *et al.*, 1977). The morphological appearances of these tumours can be variable and their histopathological diagnosis is sometimes difficult, especially in the absence of cross striations. Diagnosis is then based either on immunocytochemical staining for a specific tumour marker or on electron microscopy. As the latter is expensive and time-consuming and requires the availability of an electron microscope, these tumours are generally first subjected to immunocytochemical analysis. The expression of myoglobin by these sarcomas is therefore regarded as a useful diagnostic marker (Corson & Pinkus, 1981; Kindblom *et al.*, 1982; Brooks, 1982). However, many different commercial myoglobin antisera are available for this purpose and their reported rates of reactivity show great variability. In this study we report the reactivity of a commercially available antibody to myoglobin (Dakopatts) on a large series of sarcomas. From these results we assess the usefulness of this antibody as a marker of rhabdomyosarcomas and compare its performance to other commercially available antibodies to myoglobin.

Correspondence: M. Leader, Dept. of Pathology, Beaumont Hospital, Beaumont, Dublin 9, Ireland.

Received 17 February 1988; and in revised form, 22 August 1988.

### Materials and methods

One hundred and eighty-three soft tissue sarcomas including 25 rhabdomyosarcomas, 41 carcinomas and 27 malignant melanomas were included in the study (Table I). The sarcomas were taken from the files of the Histopathology Department of the Westminster Medical School and Hospital and form part of a large study which examined the immunocytochemical profile of different types of soft tissue sarcomas (Leader *et al.*, 1986*a*,*b*,*c*, 1987*a*,*b*,*c*,*d*). They represent some of the sarcomas seen over a 20-year period in this institution. Only tumours with available blocks were used in this study. All had been formalin fixed and paraffin embedded. All sarcomas had been diagnosed by MacKenzie (1970), an acknowledged authority in soft tissue tumour diagnosis. His criteria for the diagnosis of sarcomas, including rhabdomyosarcomas, were similar to those of Enzinger & Weiss (1983). The subtypes of rhabdomyosarcomas included in the study are shown in Table II. Cross striations were not essential for a histopathological diagnosis of rhabdomyosarcoma. A representative section from each tumour was reacted with commercially available polyclonal rabbit anit human myoglobin obtained from Dakopatts. The peroxidase anti peroxidase (PAP) method was used (details available on request). Anti human myoglobin antiserum was used at a dilution of 1:200 for 60 min on pre-trypsinised (0.1% trypsin for 30 min) sections. Sections were reacted in batches of 20–30. Each batch included appropriate controls.

Each section was examined without knowledge of the H and E diagnosis and the results were tabulated. A positive result was interpreted as discrete granular cytoplasmic staining in tumour cells. Necrotic cells were disregarded in this evaluation.

### Results

The results are summarised in Table I and those of the rhabdomyosarcomas are shown in more detail in Table II. Tumour cells in 15 of the 25 rhabdomyosarcomas stained (Figure 1) and two results were equivocal. All the main subtypes of rhabdomyosarcomas were represented amongst the positive cases (5/7 alveolar, 7/11 embryonal, 3/5 pleomorphic). Staining in all cases was granular and restricted to the cytoplasm; membrane staining was not particularly intense. Cross striations were highlighted by the staining. The number of positively staining cells in each tumour

**Table I** Anti-myoglobin staining of sarcomas, carcinomas and melanomas

| *Tumours* | *No.* | *+ve* | *−ve* | *Equivocal* |
|---|---|---|---|---|
| Rhabdomyosarcomas | 25 | 15 | 8 | 2 |
| Leiomyosarcomas | 21 | 1 | 20 | 0 |
| Synovial sarcomas | 19 | 0 | 19 | 0 |
| Angiosarcomas | 6 | 0 | 6 | 0 |
| Clear cell sarcomas | 5 | 0 | 5 | 0 |
| Neurofibrosarcomas | 17 | 0 | 17 | 0 |
| Epithelioid sarcomas | 7 | 0 | 7 | 0 |
| Liposarcomas | 23 | 0 | 23 | 0 |
| Malignant haemangiopericytomas | 14 | 0 | 14 | 0 |
| Malignant fibrous histiocytomas | 23 | 0 | 23 | 0 |
| Fibrosarcomas | 18 | 0 | 18 | 0 |
| Sarcomas unclassified | 5 | 1 | 4 | 0 |
| Carcinomas | 31 | 0 | 31 | 0 |
| Oat cell carcinomas | 10 | 0 | 10 | 0 |
| Melanomas | 27 | 0 | 27 | 0 |

varied considerably, among the different cases. When the positively staining cells were few in number, the positive cells were randomly located rather than being arranged in clumps. The intensity of staining also varied from case to case but it has been our experience that intensity of immunocytochemical staining can vary with room temperature and with different batches of antisera and diaminobenzidine. Surprisingly, well differentiated rhabdomyoblasts with cross striations failed to stain in four instances. Conversely two pleomorphic rhabdomyosarcomas in which cross striations could not be found at light microscopy stained with anti-myoglobin. It is evident from this study that by using either the presence of cross striations or reactivity with Dakopatts myoglobin antisera as criteria for the diagnosis of rhabdomyosarcomas, the diagnosis of these tumours can be confirmed in an increased number of cases (see Table II). There were only two positive reactions amongst the 183 non-rhabdomyosarcomatous sarcomas, a leiomyosarcoma and an undifferentiated sarcoma (Figures 2 and 3). The leiomyosarcoma was a spindle cell tumour with eosinophilic cytoplasm and myofilaments were identified on PTAH staining. No cross striations were evident. Staining for desmin was positive. The undifferentiated sarcoma was a high grade sarcoma with no differentiating features morphologically. However, staining for desmin (in a retrospective study) was positive, supporting a diagnosis of a tumour showing muscle differentiation. Faint, cytoplasmic staining was also seen in squamous epithelium, in neutrophils, in bronchial epithelium, in the mucus of salivary glands and in the endothelial lining of some blood vessels in tissue invaded by the rhabdomyosarcomas. However, no staining was seen in either the carcinomas or melanomas.

### Discussion

Myoglobin is a heme protein with a molecular weight of 17,800 daltons which is found exclusively in striated muscle. Immunohistochemistry shows its localisation in regions near the sarcolemmal membrane. As its concentration varies with fibre type, immunohistochemical staining of normal muscle shows a chequerboard pattern. Caution is required in the histopathological interpretation of myoglobin staining. Positively staining residual myocytes in tissue sections must not be misinterpreted as tumour cells. Reactive histiocytes and even carcinomas, lymphomas and malignant melanomas when infiltrating skeletal muscle may show a positive reaction for myoglobin (Eusebi *et al.*, 1984). It is suggested by the authors that myoglobin may be transferred from skeletal muscle to the infiltrating tumour cells.

The results of this study using antiserum from Dakopatts show that only 60% of rhabdomyosarcomas stained (Table I). So as to exclude the possibility of incorrect diagnosis, the original histopathological diagnosis of the rhabdomyosarcomas in this study was reviewed. In each the diagnosis was confirmed. All negatively staining rhabdomyosarcomas were restained using similar trypsinisation to the initial study and a similar dilution of primary antisera. No additional staining was seen. Seven papers have examined the staining reactions of anti human myoglobin antisera with rhabdomyosarcomas (Table III) (Corson & Pinkus, 1981; Brooks, 1982; Kindblom *et al.*, 1982; Tsokos *et al.*, 1983; Kahn *et al.*, 1983; Kagawa *et al.*, 1983; de Jong *et al.*, 1984). The results in three of the seven suggest that myoglobin is a useful marker of skeletal muscle differentiation (Corson & Pinkus, 1981; Brooks, 1982; Kindblom *et al.*, 1982). However, three papers report positive staining only in 47, 36 and 50% of rhabdomyosarcomas (de Jong *et al.*, 1983; Kahn *et*

**Table II** Anti-myoglobin staining of rhabdomyosarcomas

| *Age* | *Site* | *Cross striations* | *Type* | *Anti-myoglobin* |
|---|---|---|---|---|
| 3 | Lymph node | + | embryonal | + |
| 44 | Buttock | + | embryonal | − |
| 10 | Lymph node | − | embryonal | − |
| 10 | Jaw | + | embryonal | + |
| 2 | ? | + | embryonal | − |
| 15 | Paratesticular | + | embryonal | + |
| 15 | Mouth | + | embryonal | − |
| 3 | Bladder | + | embryonal | + |
| 27 | Nose | + | embryonal | + |
| 19 | Ear | + | embryonal | + |
| 16 | Paratesticular | + | embryonal | + |
| 26 | Leg | − | alveolar | + |
| 14 | Arm | − | alveolar | − |
| 32 | Thigh | + | alveolar | − |
| ? | ? | − | alveolar | + |
| 24 | Palate | + | alveolar | + |
| 24 | Leg | − | alveolar | + |
| 25 | Hand | + | alveolar | + |
| 85 | Arm | + | pleomorphic | E |
| 84 | Leg | + | pleomorphic | + |
| ? | Thigh | − | pleomorphic | + |
| 66 | Mouth | − | pleomorphic | − |
| 28 | Thigh | − | pleomorphic | + |
| 23 | Thigh | + | mixed | E |
| 14 | Thigh | − | mixed | − |

E = equivocal.

**Figure 1** A rhabdomyosarcoma showing undifferentiated small round tumour cells and also some well-differentiated rhabdomyoblasts. The latter (arrows) have stained with the myoglobin antisera. Anti-myoglobin ×400.

**Figure 2** A leiomyosarcoma that stained with anti-myoglobin. The tumour is formed by spindle and ovoid cells arranged in fascicles. There is no evidence of cross striations or of strap shaped cells. H and E ×400.

**Figure 3** An undifferentiated sarcoma that stained with anti-myoglobin. The tumour is formed by ovoid and round cells with vesicular nuclei and prominent nucleoli. Some cells had tapering cytoplasm (arrow) suggestive of strap cells but no cross striations were identified. A heavy polymorphic infiltrate was seen focally in the tumour, as shown here, but was absent in other areas. The differential diagnosis included a malignant inflammatory fibrous histiocytoma and a rhabdomyosarcoma. H and E ×400.

*al.*, 1983; Kagawa *et al.*, 1983) respectively. In another study it was found to be a useful marker of alveolar but not of embryonal rhabdomyosarcomas; no pleomorphic rhabdomyosarcomas were included in the study (Tsokos *et al.*, 1983).

This review of the literature showing reported rates of positive staining ranging from 100 to 36% in different series (Table III) highlights the disparity in the documented staining reactions of rhabdomyosarcomas and myoglobin antiserum. The most likely reason for this disparity is the differing source of antibody. It is noteworthy that 3/5 studies using antiserum from Cappel Laboratories attained a high rate of reactivity (76, 88 and 100%). Other possible causes for the disparity include variability of fixation of tissues and possibly a difference in enzyme digestion of tissue sections but the reports do not include sufficient details to confirm this. With regard to the positive reactions in the leiomyosarcoma and unclassified sarcoma in this study, it was of interest that both tumours had been earlier treated by radiotherapy. It has been previously suggested that radiotherapy may give misleading results with immunocytochemical tumour markers (Leader *et al.*, 1986*b*, 1987*a*). The reason for this is unclear; it may be that the cells 'mop up' the primary antibody non-specifically. This is suggested by positivity in irradiated viable skeletal muscle using anti factor VIII related antigen in an earlier study (Leader, 1986*b*). It may also be related in some instances to the induction of a new line of differentiation by radiotherapy. The undifferentiated tumour can almost certainly be reclassified as a rhabdomyosarcoma in view of its positivity for myoglobin and desmin and therefore it can be regarded as a genuine positive reaction. Therefore in this study there was only one positive tumour among 225 non-rhabdomyosarcomatous tumours.

A number of studies have also investigated the value of other markers such as myosin (Tsokos *et al.*, 1983), creatinine kinase (Tsokos *et al.*, 1983), creatinine kinase MM (Tsokos *et al.*, 1983; Kahn *et al.*, 1983), calsequestrin and calcium magnesium-dependent ATPase of sarcoplasmic reticulum (Kahn *et al.*, 1983) and desmin (Kahn *et al.*, 1983; Leader *et al.*, 1987*a*; Kias *et al.*, 1987) in the diagnosis of rhabdomyosarcomas. The first three of these appear to be more sensitive indicators of skeletal muscle differentiation than myoglobin; however, they are associated with a significant false positive reaction rate among carcinomas (approximating 30%) and therefore their discriminatory value is limited. Desmin, ATPase and calsequestrin were found in a higher percentage of rhabdomyosarcomas than myoglobin, but the specificity of only desmin has been investigated and this in only one study (Leader *et al.*, 1987*a*).

Tumour markers have a useful role in diagnostic surgical pathology. Positive staining must be carefully interpreted and a positive result accepted only when the tumour cells are viable, not adjacent to foci of tumour necrosis and when the staining is discrete, granular and confined to or within the cell. When one uses these criteria for a positive reaction and when one uses appropriate positive and negative controls, positivity in a cell reflects the presence within the cell of an epitope to which the antibody has been raised. This result therefore can be used to indicate that the tumour cell shows a specific line of differentiation when the epitope being recognised is specific. Of course it must be borne in mind that, with polyclonal antisera in particular, cross reactivity may be found with other types of tumours due to the variety of possible epitopes recognised by this type of antiserum.

**Table III** Other reports of anti-myoglobin staining in rhabdomyosarcomas

| Tumours | No. | +ve | −ve | Antibody source | Cross striations | Authors |
|---|---|---|---|---|---|---|
| Alveolar | 7 | 5 | 2 | Cappel | 1 | Corson & Pinkus (1981) |
| Embryonal | 5 | 5 | 0 | | 3 | |
| Pleomorphic | 5 | 3 | 2 | | 1 | |
| Alveolar | 3 | 3 | 0 | Cappel | n.k. | Brooks (1982) |
| Embryonal | 15 | 12 | 3 | | | |
| Pleomorphic | 9 | 9 | 0 | | | |
| Alveolar | 9 | 9 | 0 | Cappel | n.k. | Kindblom *et al.* (1983) |
| Embryonal | 9 | 9 | 0 | | n.k. | |
| Alveolar | 8 | 4 | 4 | Cappel | n.k. | de Jong *et al.* (1984) |
| Embryonal | 12 | 5 | 7 | | | |
| Pleomorphic | 3 | 2 | 1 | | | |
| Alveolar | 11 | 10 | 1 | Cappel | n.k. | Tsokos *et al.* (1983) |
| Embryonal | 11 | 5 | 6 | | | |
| Alveolar | 12 | 8 | 4 | Behring | n.k. | Kahn *et al.* (1983) |
| Embryonal | 53 | 16 | 37 | | | |
| Alveolar | 12 | 4 | 8 | Miles-Yede | 1 | Kagawa *et al.* (1983) |
| Embryonal | 9 | 6 | 3 | | 3 | |
| Pleomorphic | 5 | 3 | 2 | | 4 | |

n.k. = not known.

The pathologist should also be aware that radiotherapy may give misleading results on antibody staining as already outlined.

In conclusion this paper demonstrates that antimyoglobin is a specific marker of rhabdomyosarcomas, showing positive reactivity in only 0.4% of non-rhabdomyosarcomatous sarcomas. However, its sensitivity as a tumour marker is variable, being dependent at least on the source of the antibody. Before the histopathologist places too much emphasis on the results obtained with tumour markers it is essential that their sensitivity and specificity are known from investigations such as this.

Dakopatts kindly supplied the primary antiserum used in this study and Amersham International kindly provided financial assistance. Mary Leader is in receipt of a grant from the North West Thames Area Health Authority and from the Westminster Hospital Research Trust. We are very grateful to Professor D.H. MacKenzie, who very kindly allowed us full access to his collection of sarcomas, without which this study could not have been undertaken.

### References

BROOKS, J.J. (1982). Immunohistochemistry of soft tissue tumours. Myoglobin as a tumour marker for rhabdomyosarcoma. *Cancer*, **50**, 1757.

CORSON, J.M. & PINKUS, G.E. (1981). Intracellular myoglobin. A specific marker for skeletal muscle differentiation in soft tissue sarcomas. An immunoperoxidase study. *Am. J. Pathol.*, **103**, 384.

DE JONG, A.S., VAN-VARK, M., ALBUS-LUTTER, C.E., VAN-RAAMSDONK, W. & VOUTE, P.A. (1984). Myosin and myoglobin as tumour markers in the diagnosis of rhabdomyosarcoma. A comparative study. *Am. J. Surg. Pathol.*, **8**, 521.

ENZINGER, F.M. & WEISS, S.H. (1983). *Soft Tissue Tumours*. C.V. Mosby: St Louis.

EUSEBI, V., BONDI, A. & ROSAI, J. (1984). Immunohistochemical localization of myoglobin in nonmuscular cells. *Am. J. Surg. Pathol.*, **8**, 51.

KAGAWA, N., SANO, T., INABA, H., MORI, K. & HIZAWA, K. (1983). Immunohistochemistry of myoglobin in rhabdomyosarcomas. *Acta. Pathol. Jpn.*, **33**, 515.

KAHN, H.J., YEGER, H., KASSIM, O. & 5 others (1983). Immunohistochemical and electron microscopic assessment of childhood rhabdomyosarcoma. Increased frequency of diagnosis over routine histologic methods. *Cancer*, **51**, 1897.

KIAS, P., KUMAR, P., MARSDEN, H.B. & 4 others (1987). Evaluation of desmin as a diagnostic and prognostic marker of childhood rhabdomyosarcomas and embryonal sarcomas. *Br. J. Cancer*, **56**, 361.

KINDBLOM, L., SEIDAL, T. & KARLSSON, K. (1982). Immunohistochemical localization of myoglobin in human muscle tissue and embryonal and alveolar rhabdomyosarcoma. *Acta. Pathol. Microbiol. Immunol. Scand.*, **90**, 167.

LEADER, M., COLLINS, M., PATEL, J. & HENRY, K. (1986*a*). Anti neuron specific enolase staining reactions in sarcomas and carcinomas: Its lack of neuroendocrine specificity. *J. Clin. Pathol.*, **39**, 1186.

LEADER, M., COLLINS, M., PATEL, J. & HENRY, K. (1986*b*). Staining for factor VIII related antigen and *Ulex europaeus* agglutinin 1 (UEA-1) in 230 tumours. An assessment of their specificity for angiosarcomas and Kaposi's sarcomas. *Histopathology*, **10**, 1153.

LEADER, M., PATEL, J., MAKIN, C.A.M. & HENRY, K. (1986*c*). An analysis of the sensitivity and specificity of the cytokeratin marker CAM 5.2 for epithelial tumours. *Histopathology*, **10**, 1315.

LEADER, M., COLLINS, M., PATEL, J. & HENRY, K. (1987*a*). Desmin: Its value as a marker of muscle derived tumours using a commercial antibody. *Virchows Archiv.*, **411**, 345.

LEADER, M., COLLINS, M., PATEL, J. & HENRY, K. (1987*b*). Vimentin: An evaluation of its role as a tumour marker. *Histopathology*, **11**, 63.

LEADER, M., COLLINS, M., PATEL, J. & HENRY, K. (1987*c*). Synovial sarcoma: True carcinoma-sarcoma? *Cancer*, **59**, 2096.

LEADER, M., PATEL, J., COLLINS, M. & HENRY, K. (1987*d*). Anti-alpha-1-antichymotrypsin staining of 194 sarcomas and 38 carcinomas and 17 malignant melanomas. *Am. J. Surg. Pathol.*, **11**, 133.

MACKENZIE, D.H. (1970). *The Differential Diagnosis of Fibroblastic Disorders*. Blackwell Scientific: Oxford.

RUSSEL, W.O., COHEN, H.J., ENZINGER, F.M. & 7 others (1977). A clinical and pathological staging system for soft tissue sarcomas. *Cancer*, **40**, 1562.

TSOKOS, M., HOWARD, R. & COSTA, J. (1983). Immunohistochemical study of alveolar and embryonal rhabdomyosarcoma. *Lab. Invest.*, **48**, 148.